# Integrated pan-cancer and scRNA-seq analyses identify a prognostic coagulation-related gene signature associated with tumor microenvironment in lower-grade glioma

**DOI:** 10.1007/s12672-024-01114-w

**Published:** 2024-07-02

**Authors:** Xuehuan Wen, Songjie Bai, Zuochun Fang, Weiguo Zhu

**Affiliations:** 1 Department of Oncology, The Affiliated Cangnan Hospital, Wenzhou Medical University, Wenzhou, 325800 Zhejiang China; 2 Department of Cardiovascular Surgery, The First Affiliated Hospital, Jiangxi Medical College, Nanchang University, Nanchang, 330006 Jiangxi China; 3 Department of Critical Care Medicine, Longgang People’s Hospital, Wenzhou, 325800 Zhejiang China; 4 Department of Cardiology, Xinhua Hospital Affiliated to Shanghai Jiao Tong University School of Medicine, Shanghai, 200092 People’s Republic of China

**Keywords:** Pan-cancer, Coagulome, Lower-grade glioma, Prognostic signatures, Tumor microenvironment

## Abstract

Cancer-associated thrombosis is a significant complication in cancer patients, leading to increased morbidity and mortality. The expression of coagulation/fibrinolysis genes, termed the "coagulome", plays a critical role in this process. Using the single-sample gene set enrichment analysis (ssGSEA), we identified seven cancer types with significantly activated coagulation pathways, focusing on lower-grade glioma (LGG) and stomach adenocarcinoma due to their predictive value for overall survival. Through 1000 iterations of the Least Absolute Shrinkage and Selection Operator (LASSO), we selected prognostic genes and constructed effective Cox regression models, particularly for LGG. Incorporating clinical characteristics, we constructed a nomogram for LGG, achieving an impressive area under the curve (AUCs) of 0.79, 0.82, and 0.81 at 1, 3, and 5 years in the test dataset, indicating strong potential for clinical application. Functional enrichment analysis between high-risk and low-risk LGG groups revealed significant enrichment of genes involved in the inflammatory response, interferon-gamma response, and epithelial-mesenchymal transition pathways. Combined with CIBERSORT and single-cell RNA sequencing analysis of LGG, our results demonstrated that the interplay between coagulation and the tumor microenvironment, particularly involving gliomas and myeloid cells, significantly influences tumor progression and patient outcomes.

**Supplementary Information:**

The online version contains supplementary material available at 10.1007/s12672-024-01114-w.

## Introduction

Cancer-associated thrombosis (CAT), a major complication in cancer patients, encompasses venous thromboembolism (VTE), arterial events, and subclinical hypercoagulable states. It significantly increases mortality and morbidity rates [1, 2]. A study highlighted that cancer-related VTE patients had the highest mortality rate of 45.3 per 100 person-years, compared to disease-free participants (0.63), VTE-only (5.0), and cancer-only patients (9.2) [3]. CAT presents distinctive features that differentiate it from ordinary VTE cases [4]. Moreover, varying cancer types display divergent CAT ratios, with tumors of the bone, ovary, brain, and pancreas associated with the highest incidence [5], suggesting the intricate mechanisms of CAT.

The mechanisms underlying CAT are multifaceted. Numerous studies indicate that the CAT risk in patients is influenced by a confluence of patient-related factors (age, comorbidities, VTE history, etc.), cancer-related factors (tumor location, histology, stage, time since diagnosis, etc.), and treatment-related factors (surgery, hospitalization, chemotherapy, central venous catheters, etc.) [6–8]. Aside from external factors, the main mechanisms of CAT involve tumor cells expressing hemostatic proteins, the release of microparticles, inflammatory cytokines, and proangiogenic factors by tumor and/or host cells, as well as the expression of adhesion molecules that bind to platelets, endothelial cells, and leukocytes [9]. It is becoming evident that CAT is not merely a secondary effect of tumor growth but is directly connected to the molecular characteristics of cancer cells and their genetic and epigenetic oncogenic drivers [2]. Furthermore, scientists propose that the expression of coagulation/fibrinolysis genes across different primary tumor types, termed the "coagulome", plays a pivotal role in CAT [10].

In a breast cancer study, several coagulome factors correlated with tumor microenvironment (TME) characteristics and were expressed by nonmalignant cells in the TME. Notably, the PROCR-THBD-PLAT signature demonstrated a promising predictive value for combination chemotherapy with fluorouracil, epirubicin, and cyclophosphamide [11]. Another study explored the genes F3, PLAU, PLAT, PLAUR, SERPINB2, and SERPINE1 across 32 cancer types using The Cancer Genome Atlas (TCGA) data. It revealed that high expression of the fibrinolysis gene cluster (PLAU, PLAUR, SERPINE1) was consistently linked to TME characteristics, such as monocytic infiltration and high expression of key immune response checkpoints [12]. Furthermore, in glioblastoma multiforme, local microthrombosis and peripheral venous thromboembolism are closely associated with oncogenic driver mutations and their impact on coagulome expression, with different molecular subtypes showing distinct coagulation gene expression profiles [13]. These studies highlight the coagulome's critical and multifaceted role in cancer. However, there is still a lack of systematic studies examining the coagulome's impact on tumor prognosis across various cancers.

In this study, we performed single sample gene set enrichment analysis (ssGSEA) analysis using the gene set positive regulation of coagulation (GO:0050820) across 33 tumor types in TCGA and Genotype-Tissue Expression (GTEx). The Kaplan–Meier (KM) survival analysis revealed that the overall survival (OS) rates of lower-grade glioma (LGG) and stomach adenocarcinoma (STAD) were most significantly correlated with the ssGSEA score, showing the lowest p-values. We then constructed a prognostic signature for LGG and STAD with a strong area under the curve (AUC). Further analysis focused on LGG, where the high-risk group showed greater enrichment in interferon response, inflammatory response and epithelial-mesenchymal transition (EMT) pathways. Additionally, single-cell RNA sequencing (scRNA-seq) analysis of LGG indicated that the genes in the signature were predominantly highly expressed in glioma and myeloid cells.

## Methods

### Data source and preprocessing

The bulk RNA-sequencing data of 33 types of cancers were sourced from TCGA (https://portal.gdc.cancer.gov/), and the related RNA-seq data of normal tissue were downloaded from GTEx (https://gtexportal.org/home/). The validation of RNA-seq data of LGG was downloaded from Chinese Glioma Genome Atlas (CGGA) (http://www.cgga.org.cn/), and the validation data of STAD GSE83347 was downloaded from Gene Expression Omnibus (GEO) (https://www.ncbi.nlm.nih.gov/geo/). RNA-seq data of transcripts per million was log2 transformed for further analysis. Furthermore, the scRNA-seq data of LGG was downloaded from GSE182109, where the LGG samples were extracted for further study.

### Pathway activity analyses

To assess the enrichment levels of coagulation-related pathways in each sample, we referred to two previous publications [14, 15] and performed ssGSEA using the "GSVA" R package. We focused on the "GO_BP_Positive_Regulation_of_Coagulation" (GO:0050820) gene set from the Gene Ontology Biological Process (GO BP) collection. ssGSEA is a rank-based method that calculates an enrichment score for a specific gene set within an individual sample's gene expression profile.

### Survival analysis with Kaplan–Meier curve

We used RNAseq and clinical data from various TCGA projects for survival analysis across multiple cancer types. OS was assessed using the KM method, with statistical analyses and visualizations performed in R (version 4.2.3) using the survival package (version 3.5–3) for analysis and the “survminer” package (version 0.4.9) for visualization. Patients were stratified by ssGSEA scores or risk scores, and survival curves were compared using log-rank tests to determine the statistical significance of differences between groups.

### LASSO regression for feature selection and multivariate cox regression

We employed LASSO regression for feature selection to construct a robust prognostic signature, followed by multivariate Cox regression analysis. Acknowledging the potential impact of arbitrary random sample splitting on sparse high-dimensional data, we adopted a "multisplit" approach to enhance variable selection consistency while controlling finite sample error, as previously reported [16]. Specifically, we performed 1000 iterations of subsampling without replacement, each time selecting 75% of the dataset. We identified markers with a repeat occurrence frequency exceeding 300 across these iterations. The tuning parameters were determined based on the expected generalization error estimated from tenfold cross-validation and information-based criteria, such as Akaike Information Criterion (AIC) and Bayesian Information Criterion (BIC). We selected the largest lambda value such that the error was within one standard error of the minimum, a technique known as "1-se" lambda. The genes selected through this process were subsequently used as inputs for multivariate Cox regression analysis, ensuring that the final model accounted for robust predictive performance.

### Validation of model with survival ROC

To validate the Cox regression model for LGG and STAD, survival receiver operating characteristic (ROC) analysis was performed. For LGG, the TCGA-LGG dataset was used as the training data, while the CGGA-329 and CGGA-693 datasets were used as test data. The TCGA-STAD dataset served as the training data for STAD, and the GSE84437 dataset was used for testing. This approach allowed us to assess the predictive performance of our model by evaluating its accuracy in distinguishing between different survival outcomes in both training and independent test datasets.

### Construction of nomogram in TCGA-LGG

Considering that the risk model for LGG, but not STAD, was significantly associated with poor prognosis, we aimed to enhance the clinical applicability and usability of the constructed risk model. To achieve this, we developed a nomogram based on multiple clinical factors, including age, tumor grade, 1p/19q codeletion status, Isocitrate Dehydrogenase (IDH) mutation status, and risk score. This nomogram was designed to predict the probability of prognostic survival at 3 and 5 years for LGG patients, utilizing data from the TCGA-LGG dataset. The nomogram was then validated using the CGGA-329 and CGGA-693 datasets, ensuring its robustness and predictive accuracy in independent cohorts. By incorporating these key variables, the nomogram provides a comprehensive tool for personalized survival prediction in clinical settings.

### Functional enrichment analysis

To explore the differential profiles between high-risk and low-risk groups of LGG, we first performed differential expression analysis using the limma package. Genes with an absolute log fold change (|logFC|) > 1 and a false discovery rate (FDR) < 0.05 were selected for further analysis. These differentially expressed genes (DEGs) were then subjected to Gene Ontology (GO) enrichment analysis, with GO Biological Process (BP) terms clustered using the simplifyEnrichment R package [17]. Kyoto Encyclopedia of Genes and Genomes (KEGG) pathway enrichment analysis was conducted separately for upregulated and downregulated DEGs, displaying the top 6 pathways for each. Furthermore, gene set enrichment analysis (GSEA) was performed on all genes to provide a comprehensive view of transcriptomic changes.

### Immune infiltration analysis

To assess immune infiltration across LGG samples, we employed the [18] algorithm, utilizing the LM22 signature matrix as a reference. This method allowed us to deconvolute the gene expression data and estimate the relative proportions of 22 distinct immune cell types within the TME of each sample. By analyzing the immune cell composition, we aimed to gain insights into the immunological landscape and its correlation with the expression levels of 7 prognostic genes.

### Single-cell analysis of GSE182109

First, we downloaded the GSE182109 dataset from the GEO database and extracted the LGG samples. Following the Seurat workflow, the scRNA-seq data underwent rigorous quality control and preprocessing. Low-quality cells were filtered out based on the criteria of mitochondrial gene expression > 20%, detected genes < 200, and ribosomal genes > 40%. The remaining high-quality data were then normalized. Principal component analysis was performed, and the top 30 principal components were selected for dimensionality reduction using the UMAP algorithm. A clustering algorithm was applied to group the cells into distinct populations. Cell type annotations were assigned based on the expression of known cell type marker genes. Additionally, we used the “AddModuleScore” function to assess the coagulome module score across all cells.

## Results

### Elevated ssGSEA score of coagulation pathway in LGG and STAD is associated with poorer OS

**Fig. 1:**
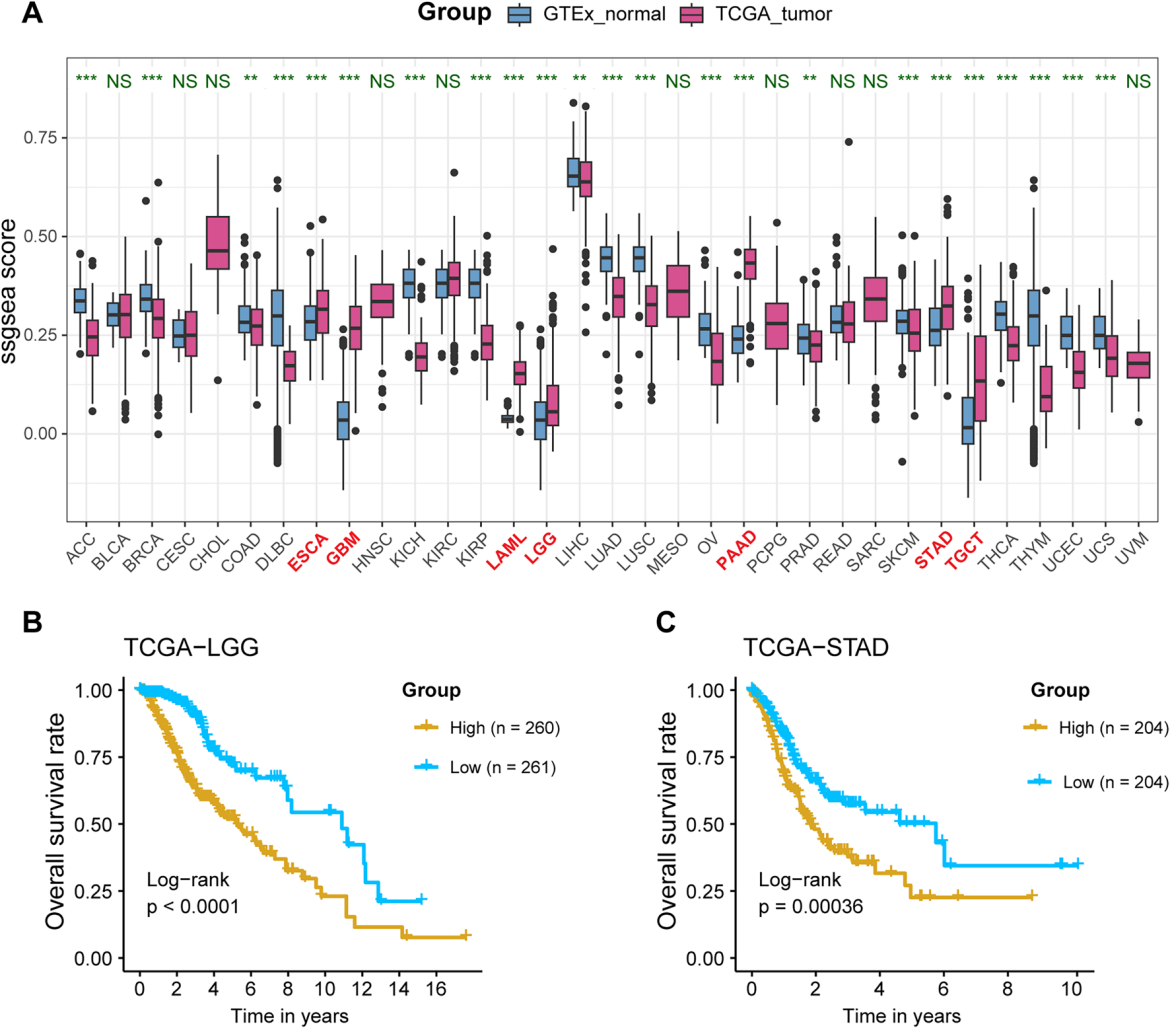
ssGSEA of coagulome across pan-cancers, normal Tissues, and its prognostic impact. **A** The ssGSEA scores for tumor samples in the TCGA dataset and normal tissue samples in the GTEx dataset. **B**-**C** The TCGA samples were divided into high and low groups based on the median ssGSEA score. **B** Kaplan–Meier (KM) survival curve for TCGA-LGG samples stratified by high and low ssGSEA scores. **C** KM survival curve for TCGA-STAD samples stratified by high and low ssGSEA scores

To evaluate the positive regulation of coagulation in tumor and normal tissues, we performed ssGSEA across 33 cancer types in TCGA and normal tissues in GTEx. The results showed a significant increase in ssGSEA scores in esophageal carcinoma (ESCA), glioblastoma multiforme (GBM), acute myeloid leukemia (LAML), LGG, STAD, pancreatic adenocarcinoma (PAAD), and testicular germ cell tumors (TGCT) compared to normal tissues (Fig. 1A). Subsequently, we conducted KM survival analysis using the median ssGSEA score to distinguish between high and low coagulation groups in these seven tumor types. Among them, LGG exhibited the most significant difference in survival between the high and low groups (Log-rank p < 0.0001, Fig. 1B), followed by STAD (Log-rank p < 0.00036, Fig. 1C). In contrast, ESCA, GBM, TGCT, LAML, and PAAD showed Log-rank p-values of 0.87, 0.24, 0.45, 0.068, and 0.61, respectively (Supplementary Fig. 1A-E).

### Construction of prognostic gene signatures for LGG and STAD

**Fig. 2:**
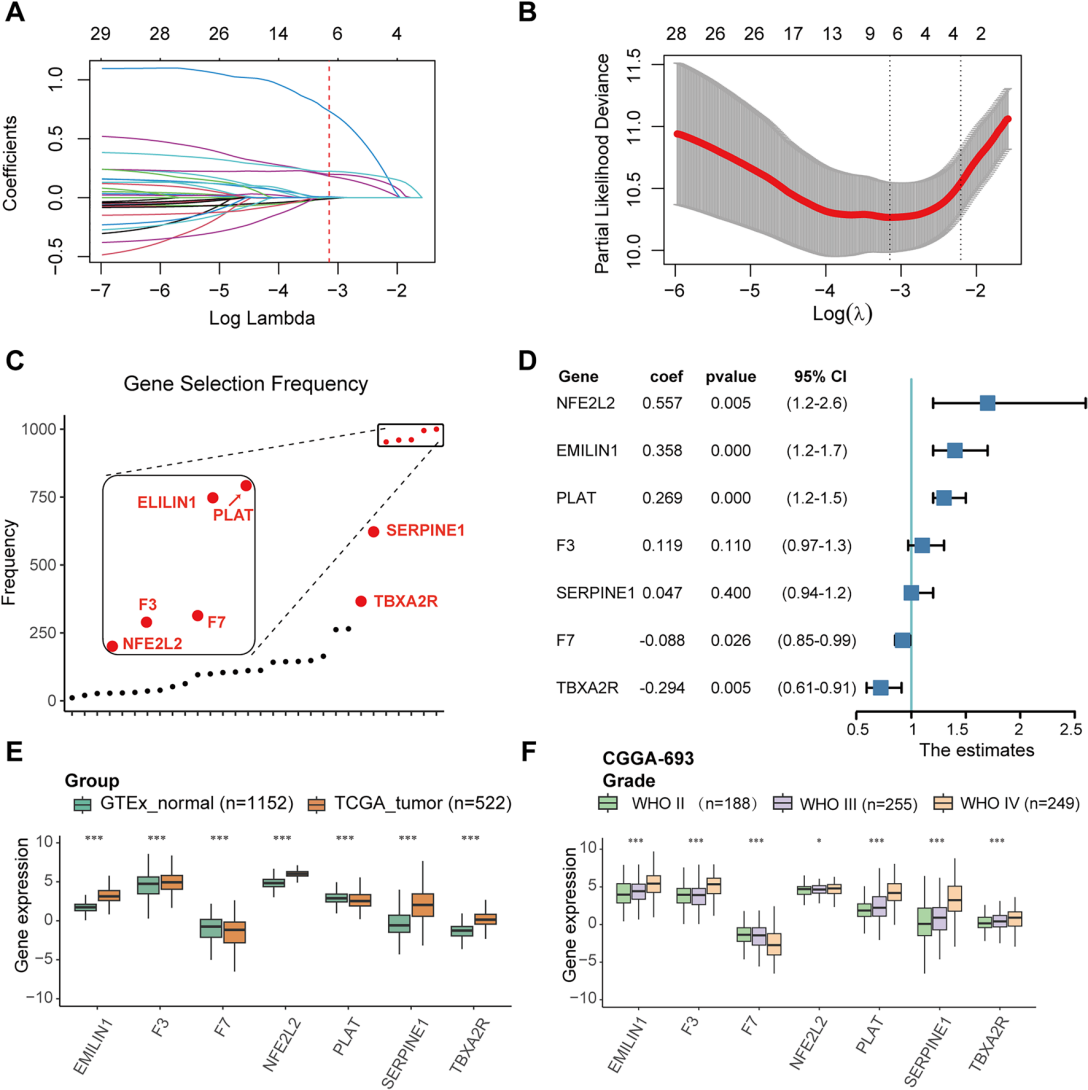
Construction of multivariate Cox regression model of LGG. **A** LASSO coefficient profiles of coagulome genes in LGG. **B** Selection of the optimal parameter (lambda) in the LASSO model, indicating the point where the cross-validated error is minimized. **C** The frequency of non-zero parameters for the 30 coagulome genes across 1000 LASSO iterations, highlighting the most frequently selected genes. **D** Forest plot showing the selected genes' *p* value*,* coefficients, and hard ratio confidence intervals from the multivariate Cox regression model. **E** Comparison of gene expression profiles between GTEx normal tissues and TCGA-LGG tumor tissues. **F** Gene expression profiles among WHO grade II, III, and IV gliomas in the CGGA-693 dataset

To construct a prognostic signature, we performed LASSO regression analysis for feature selection and multivariate Cox regression analysis to ensure robust and predictive model performance. A "multisplit" approach was employed to select prognostic gene signatures. This involved 1000 iterations of subsampling, each of which identified non-zero parameters and determined the most frequently selected genes. One iteration of the LASSO regression process is illustrated in Fig. 2A, which shows the coefficient profiles shrinking to zero as the log lambda value increases. Figure 2B displays the optimal log lambda that minimizes cross-validated error. We counted the frequency of non-zero genes, with the seven most frequently selected genes being PLAT, EMILIN1, F7, F3, NFE2L2, SERPINE1, and TBXA2R, each appearing more than 300 times (Fig. 2C).

Subsequently, we performed a multivariate Cox regression analysis. The resulting risk score formula is as follows: Risk score = (0.357 × EMILIN1 expression) + (0.119 × F3 expression) + (−0.088 × F7 expression) + (0.558 × NFE2L2 expression) + (0.268 × PLAT expression) + (0.047 × SERPINE1 expression) + (−0.294 × TBXA2R expression) The forest plot summarizes the coefficients, p-values, and 95% confidence intervals for each gene included in the model (Fig. 2D).

We then evaluated the expression profiles of these seven genes in GTEx normal tissue and TCGA tumor tissue. F7 and PLAT showed slightly lower mRNA expression levels in tumor tissues. In comparison, the other five genes exhibited significantly higher mRNA levels in tumor tissues (Fig. 2E). In the TCGA-LGG dataset, we compared the mRNA expression of the seven genes across G2 and G3 grade groups, finding significantly higher levels in G3 except for F7 (Supplementary Fig. 2A). Additionally, we validated these expression profiles in the CGGA-693 dataset, where the expression levels of six genes, excluding F7, showed a gradual increase across WHO II, WHO III, and WHO IV grades of glioma (Fig. 2F).

IDH1 is common in gliomas. A previous study suggests mutant IDH1 has potent antithrombotic activity within gliomas and peripheral circulation. Consistent with this finding, we observed that wildtype gliomas had a significantly higher risk score compared to IDH mutant gliomas (Supplementary Fig. 2B). Additionally, another study showed that the presence of 1p/19q codeletion in diffuse low-grade gliomas is significantly associated with prolonged OS and progression-free survival. The non-codeletion group had a significantly higher risk score (Supplementary Fig. 2C).

Moreover, we applied the same method to the TCGA-STAD dataset, resulting in the following risk score formula: Risk score = (0.0386 × APOH expression) + (0.0657 × CD36 expression) + (0.0989 × F2R expression) + (−0.2912 × NFE2L2 expression) + (0.0713 × PLG expression) + (0.1751 × SERPINE1 expression) + (−0.3758 × USF1 expression) + (0.0518 × VKORC1 expression) + (0.0347 × VTN expression). Among these genes, CD36 and F2R showed significant differences across G1, G2, and G3 grade groups, while the other seven genes did not exhibit significant differences (Supplementary Fig. 2D).

### Validation of prognostic signatures in LGG and STAD

**Fig. 3:**
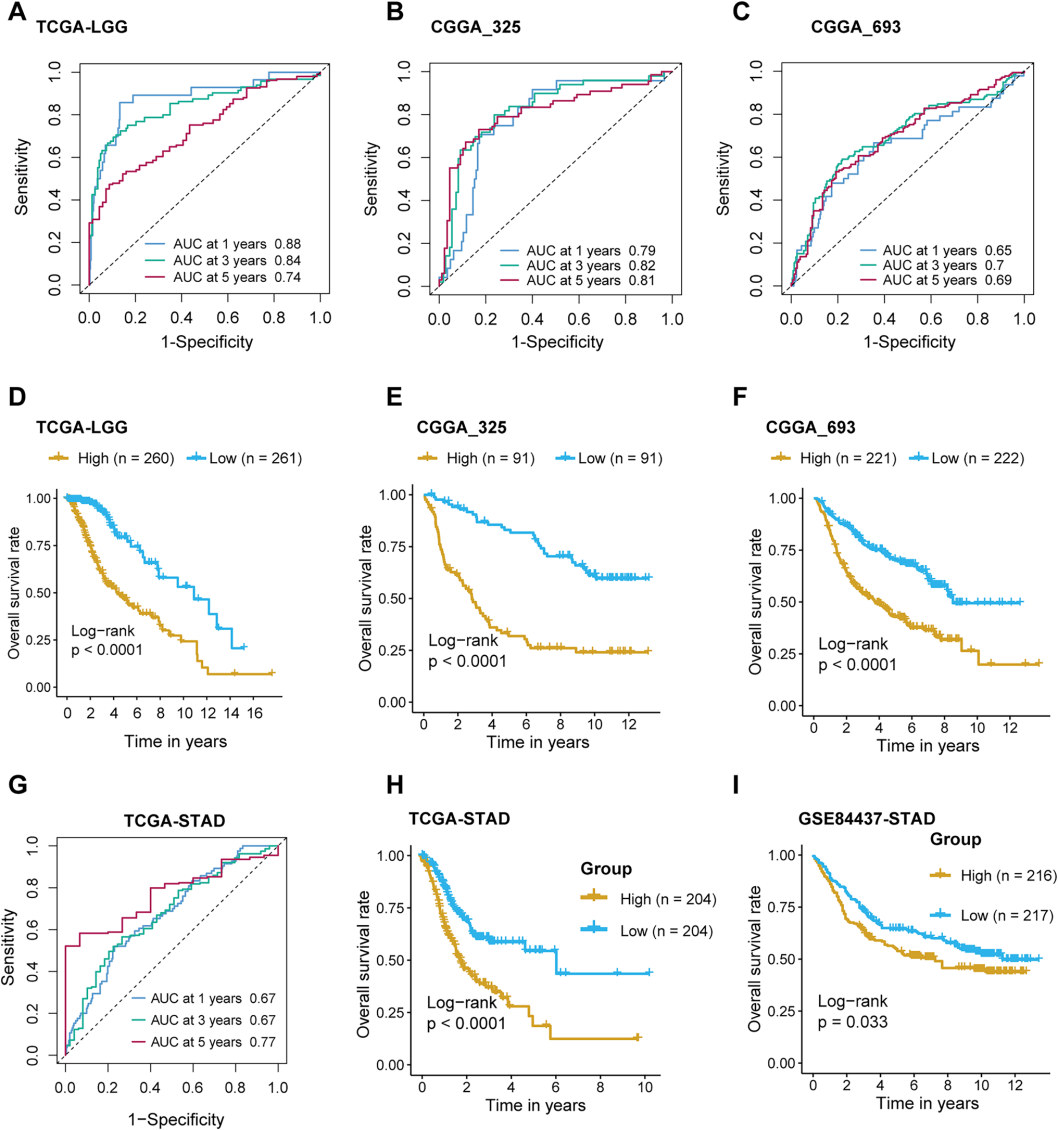
The predictive value of the Cox regression model for LGG and STAD. **A** Survival ROC curves for TCGA-LGG at 1, 3, and 5 years. **B** Survival ROC curves for CGGA-325 at 1, 3, and 5 years. **C** Survival ROC curves for CGGA-693 at 1, 3, and 5 years. **D**-**F** KM survival curves for LGG samples divided into high and low-risk groups based on the median risk score: **D** KM curve for TCGA-LGG. **E** KM curve for CGGA-325. **F** KM curve for CGGA-693. **G** Survival ROC curves for TCGA-STAD at 1, 3, and 5 years. **H**-**I** KM survival curves for STAD samples divided into high and low-risk groups based on the median risk score: **H** KM curve for TCGA-STAD. **I** KM curve for GSE84437

To evaluate the prognostic value of the signature for LGG and STAD, we performed survival ROC analysis. For the TCGA-LGG dataset, the AUC values at 1 year, 3 years, and 5 years were 0.88, 0.84, and 0.74, respectively, indicating a strong prognostic effect (Fig. 3A). We then validated the signature in the CGGA-325 and CGGA-693 datasets. In CGGA-325, the AUC values at 1 year, 3 years, and 5 years were 0.79, 0.82, and 0.81, respectively (Fig. 3B). In CGGA-693, the AUC values at 1 year, 3 years, and 5 years were 0.65, 0.70, and 0.69, respectively (Fig. 3C).

We further stratified the samples into high-risk and low-risk groups based on the median risk score. The survival analysis showed log-rank p-values below 0.0001, indicating the signature's strong predictive effect (Fig. 3D–F).

Similarly, we applied the same methods to the STAD dataset, including TCGA-STAD and GSE84437. In the TCGA-STAD dataset, the AUC values at 1 year, 3 years, and 5 years were 0.67, 0.67, and 0.77, respectively (Fig. 3G). The survival analysis for TCGA-STAD showed a log-rank p-value below 0.0001 (Fig. 3H), while in the validation dataset GSE84437, the log-rank p-value was 0.033 (Fig. 3I).

### Development and validation of a nomogram for predicting LGG prognosis

**Fig. 4:**
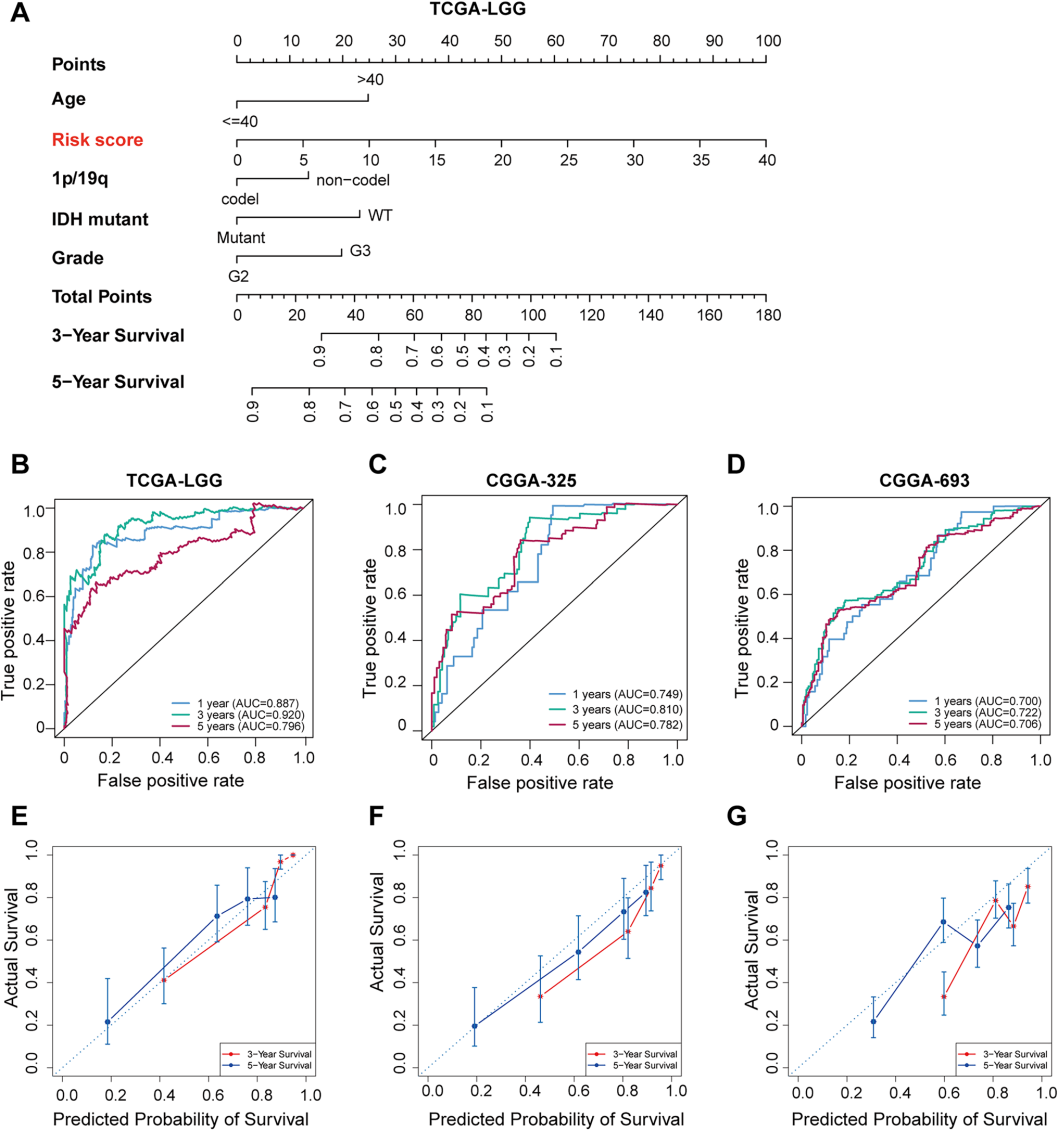
Construction and validation of a nomogram for LGG. **A** Development of the nomogram using the TCGA-LGG dataset (TCGA-LGG). **B**-**D** Survival ROC curves at 1, 3, and 5 years in training and test datasets. **B** Survival ROC curve for TCGA-LGG. **C** Survival ROC curve for CGGA-325. **D** Survival ROC curve for CGGA-693. **E**–**G** Calibration plots for training and test datasets. **E** Calibration plot for TCGA-LGG. **F** Calibration plot for CGGA-325. **G** Calibration plot for CGGA-693

Given the robust association of our LGG risk model with prognosis, particularly its strong performance in survival ROC, we chose to enhance its clinical application and usability by developing a nomogram. This nomogram incorporates age, grade, 1p/19q status, IDH mutation status, and risk score to predict the probability of 3-year and 5-year prognostic survival in LGG patients (Fig. 4A). Among these factors, the risk score had the most significant impact on predicting OS, indicating that LGG prognosis could be more accurately predicted using a risk model based on the seven selected genes (Fig. 4A).

The nomogram demonstrated predictive solid power in the TCGA-LGG dataset, with AUC values of 0.887 at 1 year, 0.92 at 3 years, and 0.796 at 5 years (Fig. 4B). Validation in the CGGA-325 dataset showed AUC values of 0.749 at 1 year, 0.81 at 3 years, and 0.782 at 5 years (Fig. 4C). Similarly, the CGGA-693 dataset showed AUC values of 0.7 at 1 year, 0.72 at 3 years, and 0.706 at 5 years (Fig. 4D). Calibration curves indicated satisfactory agreement between the predicted and observed values for the probability of 1-year, 3-year, and 5-year OS in both the TCGA-LGG (Fig. 4E) and validation datasets CGGA-325 (Fig. 4F) and CGGA-693 (Fig. 4G).

### Comparative analysis of high and low-risk patient subgroups of LGG

**Fig. 5:**
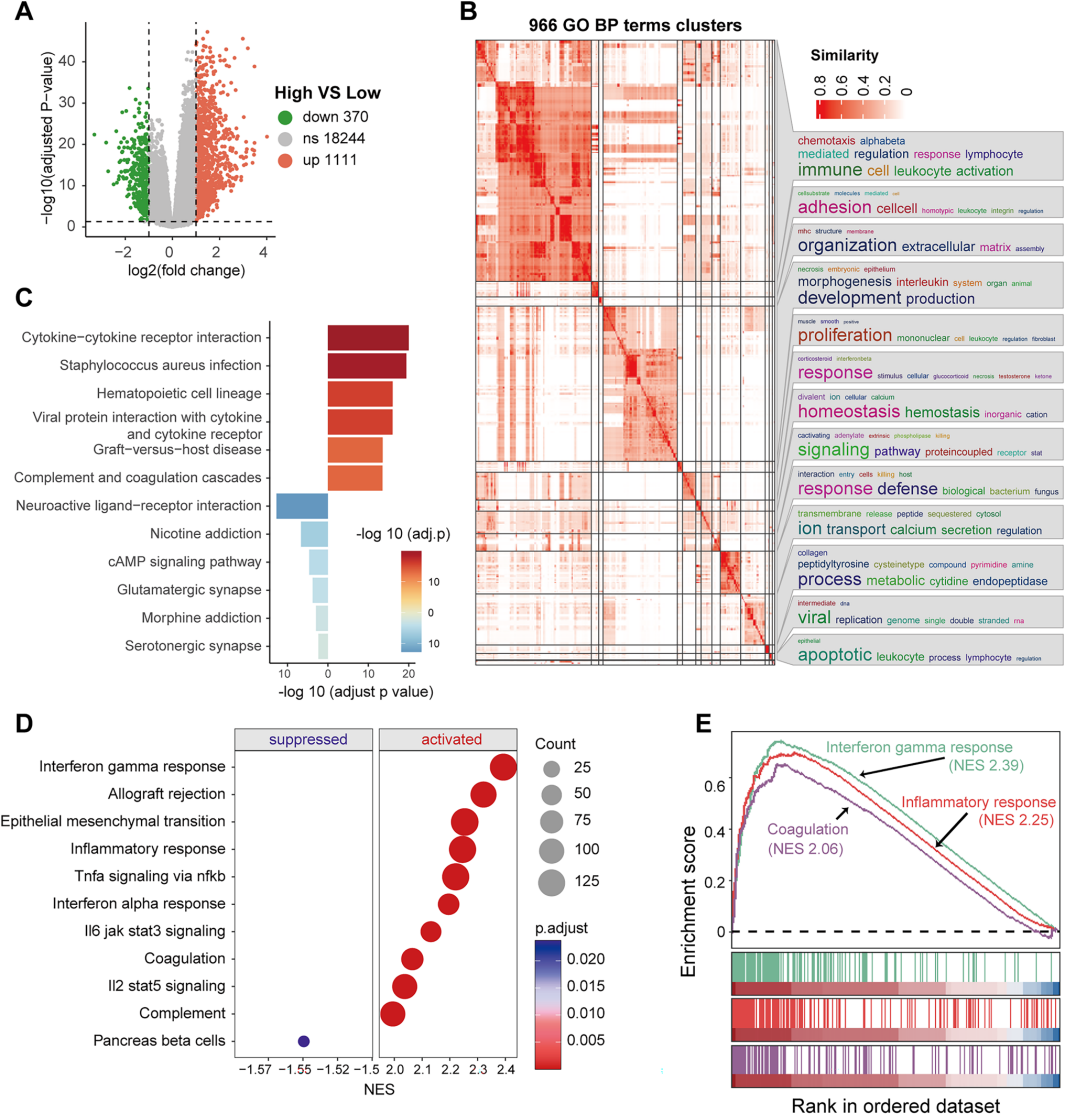
Functional enrichment analysis between high-risk and low-risk groups of LGG. **A** Volcano plot displaying DEGs, with upregulated genes in red and down-regulated genes in green. **B** Clusters of GO BP items for DEGs. **C** KEGG enrichment analysis shows the top 6 pathways for upregulated genes (in red) and down-regulated genes (in blue) separately. **D** Top 10 Gene Set Enrichment Analysis (GSEA) results for activated and suppressed HALLMARK gene sets. **E** Representative enrichment curve of GSEA results

To elucidate the biological relevance of risk scores, we identified DEGs and performed functional enrichment analyses between the high and low-risk score subgroups. Compared to the low-risk score group, the high-risk group had 1,111 upregulated genes and 370 down-regulated genes (thresholds: |log2FC|> 1 & FDR < 0.05, Fig. 5A).

GO enrichment analysis showed that the DEGs primarily involve key functional categories such as immune response, cell adhesion, development, cell proliferation, signaling pathways, defense mechanisms, ion transport, metabolism, and apoptosis (Fig. 5B). Additionally, KEGG pathway enrichment analysis revealed that upregulated genes in the high-risk group are significantly enriched in immune-related pathways, including cytokine interactions, infection response, and hematopoietic cell lineage development. In contrast, downregulated genes are enriched in pathways related to neurotransmission and receptor interactions (Fig. 5C). Consistent with these findings, GSEA indicated significant activation of immune response pathways in the high-risk score group, including interferon-gamma response, inflammatory response, coagulation, EMT, and TNF signaling. Conversely, the pancreas beta cells pathway is suppressed, indicating reduced activity related to these cells in the high-risk group (Fig. 5D, E).

In summary, these results suggest that patients in the high-risk group exhibit robust activation of immune and inflammatory responses, highlighting a potential link between immune response and coagulation in the high-risk profile.

### Association between risk score and immune cell infiltration in LGG

**Fig. 6:**
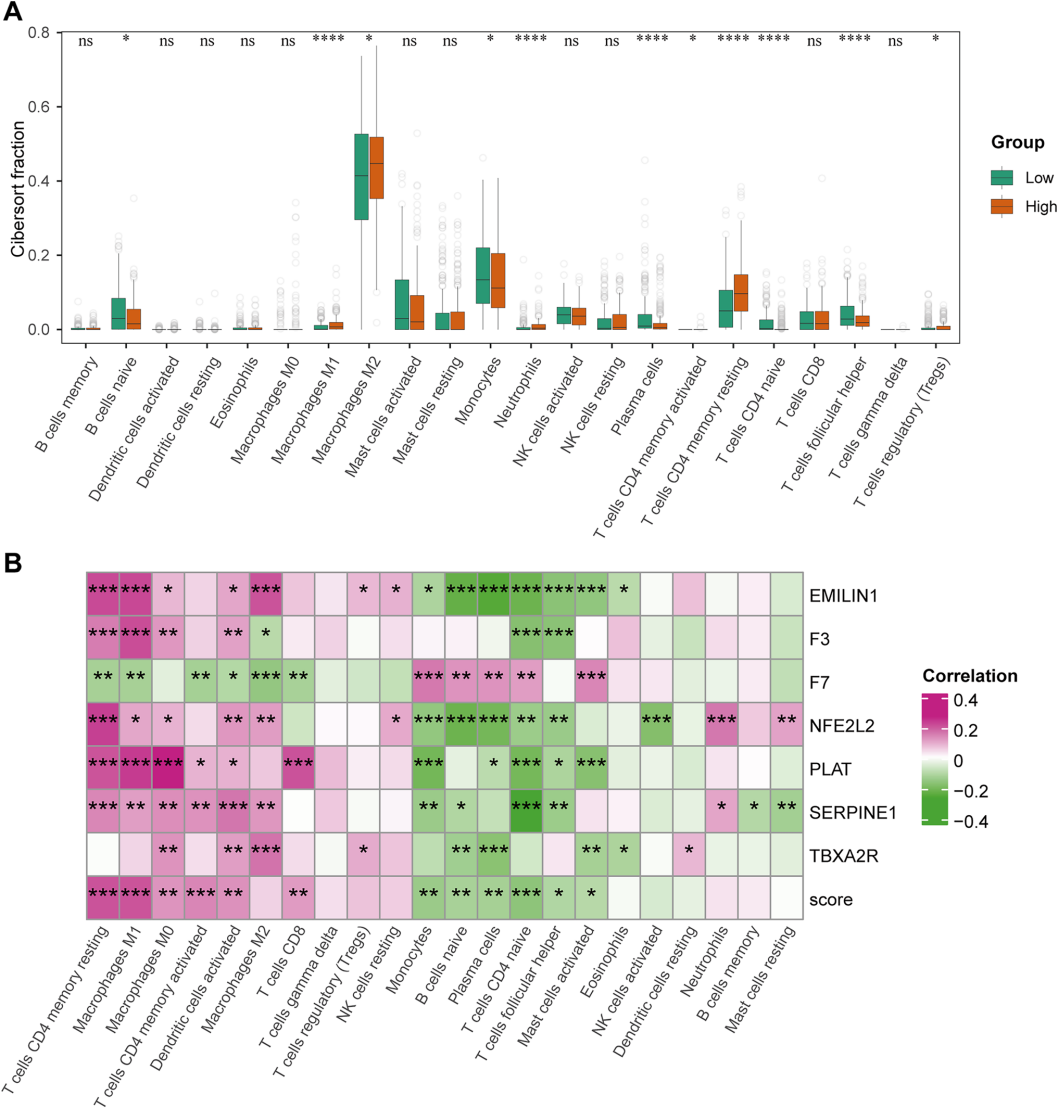
Immune infiltration in LGG. **A** An immune infiltration profile was generated using the CIBERSORT algorithm. **B** Correlation between gene expression levels and CIBERSORT fractions

Given the strong association between risk scores and immune activity, we used CIBERSORT to deconvolute the immune cell fractions in LGG patients and analyze the correlation between immune cell fractions and gene expression levels. Compared to the high-risk group, the low-risk group exhibited higher levels of immune infiltration by M1 macrophages, M2 macrophages, neutrophils, CD4 memory-resting T cells, and Tregs. In contrast, the high-risk group had higher levels of naïve B cells, monocytes, plasma cells, naïve CD4 T cells, and follicular helper T cells (Fig. 6A).

Correlation analysis between immune cell infiltration and gene expression levels revealed a global positive correlation with CD4 memory-resting T cells, CD4 memory-activated T cells, macrophages, and dendritic cells. Conversely, monocytes, naïve B cells, plasma cells, naïve CD4 T cells, and follicular helper T cells showed a global negative correlation with gene expression levels (Fig. 6B).

### Single-cell RNA sequencing analysis of LGG

**Fig. 7:**
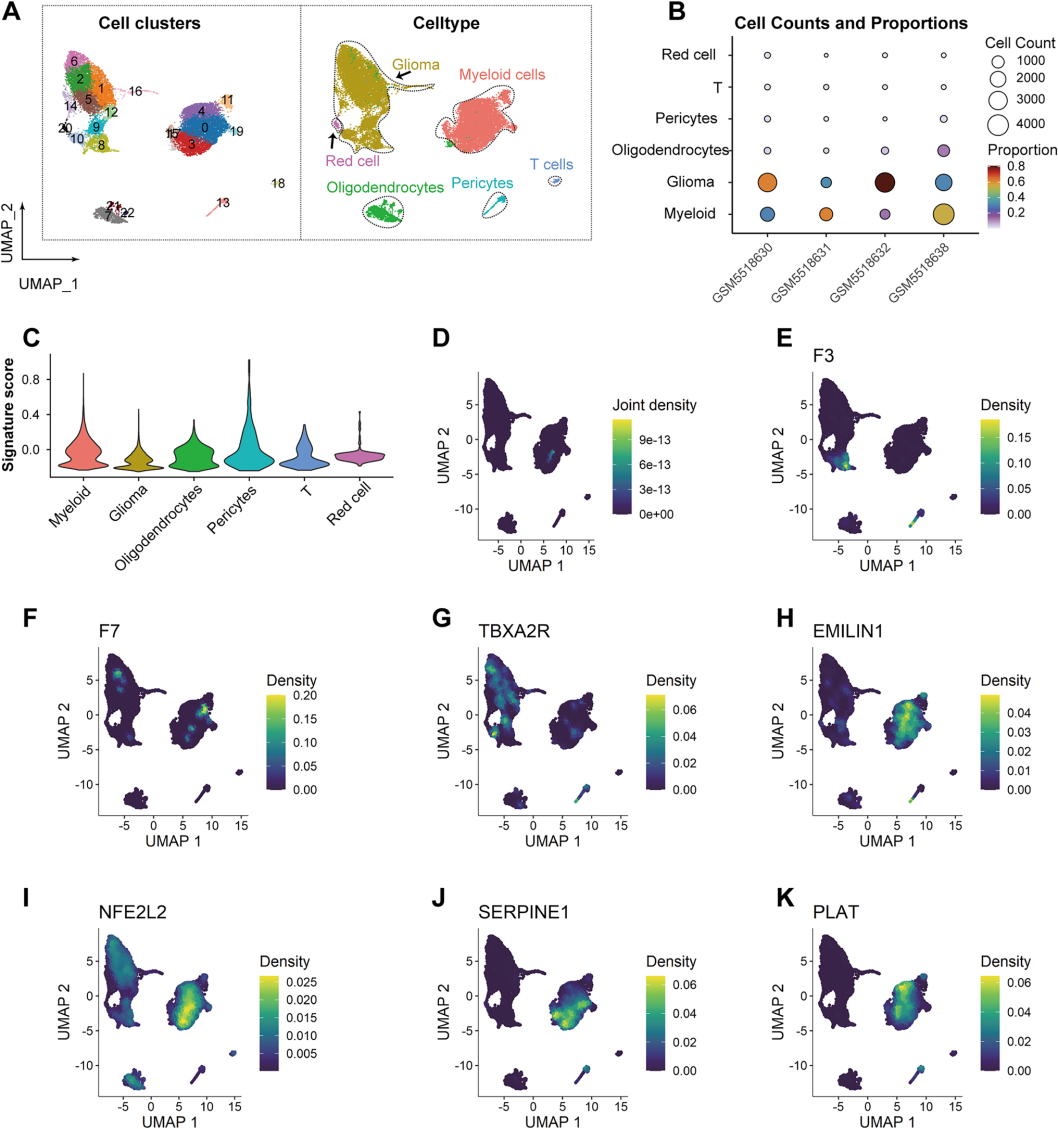
Expression profile of selected prognostic genes in scRNA-seq of LGG. **A** UMAP visualization of cell clusters (left) and cell annotations (right). **B** Cell counts and proportions of different cell types across various samples. **C** Signature scores among different cell types. **D**-**K** Density plots of selected genes and their joint density: **D** Joint density plot. **E** Density plot of F3. **F** Density plot of F7. **G** Density plot of TBXA2R. (**H**) Density plot of EMILIN1. **I** Density plot of NFE2L2. **J** Density plot of SERPINE1. **K** Density plot of PLAT

To clarify the expression profiles of the seven genes in LGG tumors, we performed single-cell RNA sequencing analysis. We identified 23 cell clusters. Based on the top positive DEGs and classical cell type markers (Supplementary Fig. 3A, B), we annotated these clusters into 6 cell types: red cells, T cells, glioma cells, oligodendrocytes, pericytes, and myeloid cells (Fig. 7A). The majority of the cells were glioma cells and myeloid cells (Fig. 7B). Using the 'AddModuleScore' function in the Seurat package, we calculated the scores of the signature genes across all cell types. The violin plot showed that myeloid cells had the highest module score (Fig. 7C).

Consistent with this result, the combined density plot of the seven genes showed that a subset of myeloid cells had the highest density (Fig. 7D). Among the individual genes, F3, F7, and TBXA2R showed higher expression densities in glioma cells (Fig. 7E–G). Conversely, EMILIN1, NFE2L2, SERPINE1, and PLAT showed higher expression densities in myeloid cells (Fig. 7H–K). These results suggest a critical role for the interaction between glioma and myeloid cells in LGG tissues.

## Discussion

This study analyzed the positive regulation of the coagulation gene set across 33 cancer types in TCGA and corresponding normal tissues in GTEx. We identified seven cancer types with significantly activated coagulation pathways. Among these, ssGSEA and KM survival analysis highlighted LGG and STAD as most correlated with OS. Using 1000 iterations of LASSO, we selected prognostic genes for LGG and STAD and constructed effective Cox regression models, particularly for LGG. The high-risk LGG group exhibited distinct biological features compared to the low-risk group, notably in inflammatory and interferon responses. CIBERSORT deconvolution and scRNA-seq analysis further demonstrated that the selected genes were mainly expressed in myeloid cells and gliomas. This systematic biology analysis highlighted the heterogeneity of coagulation profiles across various cancers. Our deeper analysis in LGG revealed the complex interactions between the TME and coagulation.

The frequency of VTE and its recurrence rate are higher in cancer patients compared to other patient groups [19]. Tumor cells can activate blood coagulation through multiple mechanisms, including procoagulant production, proinflammatory and proangiogenic cytokines release, and direct interaction with host vascular systems [20]. Behind these mechanisms, the coagulome plays a foundational role, with increasing evidence highlighting the impact of interactions between the coagulome and the TME in tumor progression [21]. Our findings indicate that coagulome activity is not uniformly upregulated across all cancer types, reflecting the intricate interplay between tumor biology and coagulation pathways. This suggests that the coagulates influence on tumor progression and patient prognosis is highly context-dependent, influenced by different cancers' unique molecular and cellular environments. Indeed, this finding is consistent with previous studies showing that the landscape of cancer coagulome expression varies significantly among different cancer types, with varying levels of tissue factor (TF), podoplanin (PDPN), inflammatory mediators, leukocytosis, and platelet counts [21–23].

Among the cancers with high coagulome activity, LGG and STAD had the strongest correlation between prognosis and the ssGSEA score, particularly LGG. However, GBM, a more malignant form of glioma, did not show a significant predictive value for the ssGSEA score (log-rank *p* = 0.24), in contrast to LGG. This discrepancy raises several potential explanations. First, the relationship between coagulation processes and tumor biology in GBM might be more intricate and complex than in LGG. Previous studies have reported morphological hallmarks of GBM, such as striking endothelial cell proliferation and occlusive intravascular thrombosis, suggesting a multifaceted interplay between coagulation and tumor biology in this aggressive cancer type [24]. Second, the more significant malignant potential of GBM could involve additional aggressive features that overshadow the prognostic impact of coagulation-related processes. Third, the higher degree of intra-tumoral heterogeneity in GBM, as revealed by single-cell sequencing studies showing significant variability in cancer cell populations' coagulates, could impact the statistical power and significance of the ssGSEA score's predictive value. This heterogeneity may further complicate the relationship between coagulome activity and prognosis in GBM [23].

In constructing the multivariate Cox regression model, we employed an unbiased feature selection strategy utilizing 1000 iterations of the LASSO algorithm. This rigorous approach aimed to identify the most robust and predictive features and enhanced the model's prognostic performance. Notably, our model outperformed a previously reported predictive model in the literature [25]. While their model achieved survival AUC values of 0.57, 0.61, and 0.65 at 1, 3, and 5 years, respectively, in the test CGGA dataset, our model exhibited superior predictive accuracy with survival AUC values of 0.79, 0.82, and 0.81 at the corresponding time points. Furthermore, we successfully applied a similar methodology to develop a predictive model for STAD, demonstrating the versatility and robustness of our approach across multiple cancer types. Notably, this STAD model also exhibited commendable predictive performance, underscoring the broad applicability of our modeling strategy.

In addition to the Cox regression models, we constructed a comprehensive nomogram that integrated clinical and molecular features, thereby capturing a more holistic representation of the predictive landscape. This integrative nomogram demonstrated exceptional predictive capability, with AUC values of 0.887, 0.92, and 0.796 at 1, 3, and 5 years, respectively, outperforming the individual models based on molecular features alone. Collectively, the superior performance compared to previous studies highlights the potential clinical utility of our models in aiding prognostic stratification and informing personalized treatment strategies for these malignancies.

The features we selected were PLAT, EMILIN1, F7, F3, NFE2L2, SERPINE1, and TBXA2R. PLAT encodes a serine protease that activates plasminogen to plasmin and has been identified as a key gene associated with immune infiltration in lower-grade gliomas [26]. EMILIN1, an extracellular matrix glycoprotein, is proposed to play a role in tumor progression and invasion in LGG [27]. F3, encoding TF, is a critical regulator of therapeutic resistance and oncogenic senescence in GBM, with a newly developed F3-targeting agent showing promising anti-tumor effects [28]. NFE2L2 (NRF2) is an important prognostic biomarker in LGGs, with higher expression levels associated with poorer outcomes and increased immune cell infiltration [29, 30]. SERPINE1 (plasminogen activator inhibitor-1, PAI-1), a serine proteinase inhibitor, has been reported as a novel biomarker for diffuse lower-grade gliomas through large-scale analysis [31]. These genes, selected through our rigorous feature selection approach, capture key regulators of coagulation, tumor progression, and immune modulation, potentially contributing to the prognostic power of our model in LGG.

Additionally, our analysis revealed that the coagulome-related model's risk score was higher in the IDH wildtype group and the non-codeletion 1p/19q group of LGG patients. These findings are noteworthy because IDH mutations and 1p/19q codeletion status are crucial molecular markers in LGGs. IDH mutations are prevalent in LGGs and confer a more favorable prognosis, serving as essential markers for classification, diagnosis, and targeted therapy development [32]. Similarly, the 1p/19q codeletion status is a vital molecular marker associated with better prognosis, increased responsiveness to specific treatments, and distinct tumor immune microenvironment characteristics [33]. These findings highlight the critical role of our coagulome-related model in stratifying LGG patients and informing personalized treatment strategies.

Although our nomogram and prognostic model demonstrate potential clinical utility, translating these findings into real-world practice presents several key challenges. External validation across diverse cohorts is necessary to establish generalisability. Addressing ethical considerations, such as data privacy and regulatory compliance, is essential when using predictive algorithms to guide treatment. Additionally, the models should complement, rather than replace, clinical judgment and shared decision-making with patients. Lastly, continuous refinement is necessary as new data emerges.

The coagulation system links immediate hemostatic responses and later inflammatory and angiogenic processes following tissue injury, a continuum often subverted in cancer [34]. Our analysis of the coagulome profile in LGG revealed significant activation of the inflammatory, interferon response, and EMT pathways in the high-risk group compared to the low-risk group, as defined by our coagulome-related model's risk score. These findings are consistent with previous studies demonstrating the intricate interplay between coagulation, inflammation, and tumor progression.

Furthermore, coagulation proteases and thrombin generation are increased in tumors, and chemotherapeutic agents can exacerbate cancer-associated thromboses. Thrombin impacts tumor cells and the immune microenvironment, promoting proinflammatory cytokine release and the accumulation of immunosuppressive cell populations [35]. Our analysis showed that the immune infiltration analysis by CIBERSORT revealed an upregulation of M2 macrophages and Tregs in the high-risk group. This aligns with thrombin's role in promoting an immunosuppressive TME by recruiting myeloid-derived suppressor cells and regulatory T cells.

Additionally, our scRNA-seq analysis identified glioma and myeloid cells as the predominant sources of coagulome-related gene expression. This supports the interplay between coagulation, inflammation, and tumor-immune interactions in LGG. Notably, M2 macrophages are known for their role in tissue repair and immunosuppression, contributing to a tumor-promoting environment. These insights highlight the coagulome's multifaceted roles in shaping an immunosuppressive and proinflammatory TME, potentially contributing to tumor progression in LGG.

Nevertheless, it is important to note that our study was not without limitations. Firstly, the primary data utilised in our analyses were derived from public repositories, which may introduce inherent biases associated with such datasets. Secondly, the sample size for the scRNA-seq data was relatively small, necessitating further validation with larger cohorts to robustly investigate the TME dynamics. Thirdly, while our findings provide valuable insights, future functional studies, including in vitro and in vivo experiments, are essential to elucidate the underlying mechanisms. Additionally, the clinical implementation of our prognostic models may face challenges, requiring further evaluation in prospective studies.

## Conclusion

The study constructed a predictive model with potential clinical applications, revealing a close relationship between coagulome genes and the immune microenvironment in LGG. These findings emphasise the importance of targeting the coagulome and its interactions with the TME when developing new therapeutic strategies for LGG treatment.

### Supplementary Information

Supplementary material 1.

Supplementary material 2.

Supplementary material 3.

## Data Availability

All data used in this study are publicly available from the following sources: TCGA database: https://portal.gdc.cancer.gov/; GTEx project: https://gtexportal.org/; GEO repository: https://www.ncbi.nlm.nih.gov/geo/; CGGA repository: (http://www.cgga.org.cn/). Accesssion numbers and detailed dataset information are provided in the methods section.
