# Supplementary figures and images for: Integrated pan-cancer and scRNA-seq analyses identify a prognostic coagulation-related gene signature associated with tumor microenvironment in lower-grade glioma
Source: Discov Oncol. 2024 Jul 2;15:256. doi: 10.1007/s12672-024-01114-w (PMC11219639; doi:10.1007/s12672-024-01114-w)

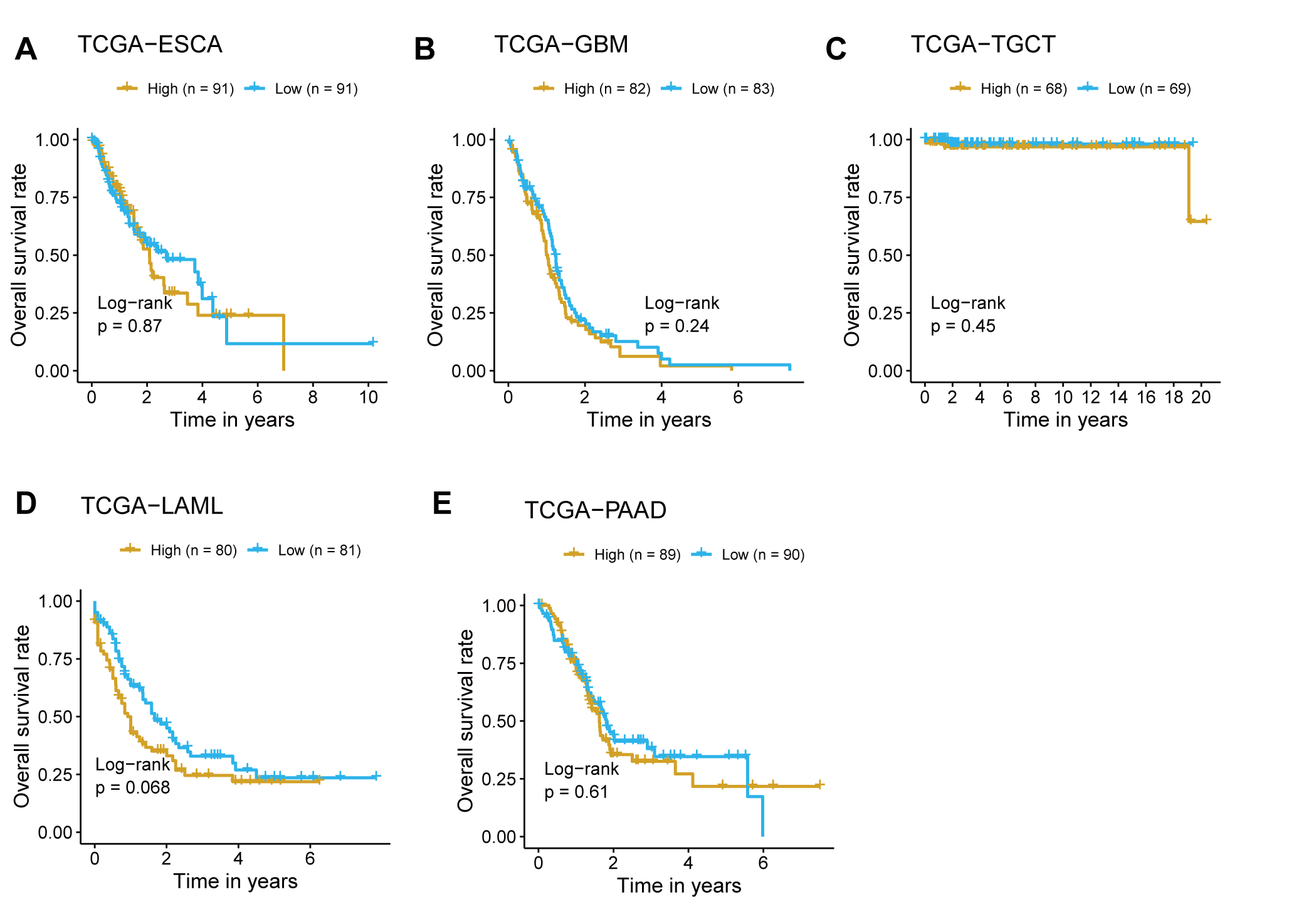

Supplement: Supplementary file 1 — Supplementary material 1. [file 12672_2024_1114_MOESM1_ESM.tif]

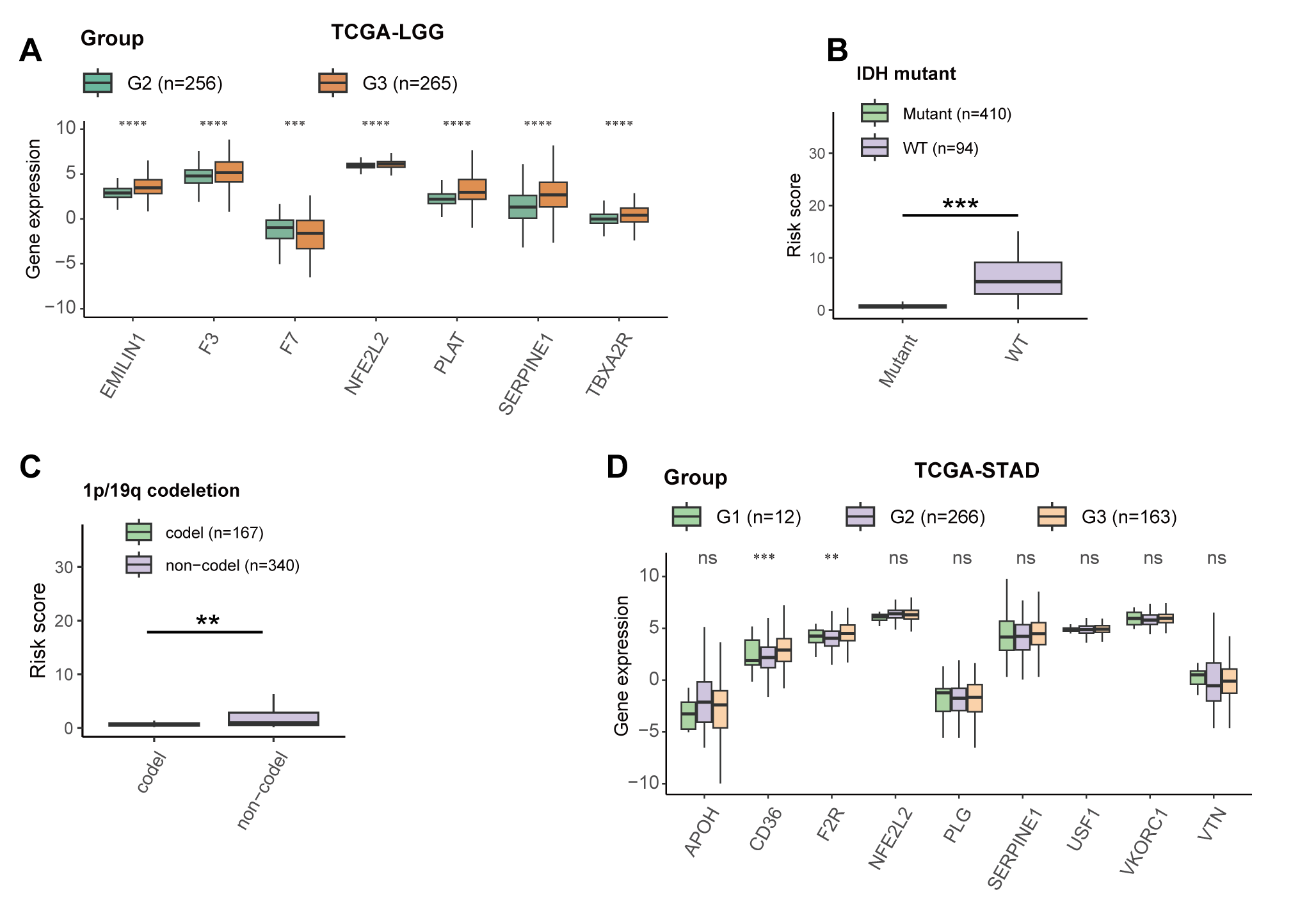

Supplement: Supplementary file 2 — Supplementary material 2. [file 12672_2024_1114_MOESM2_ESM.tif]

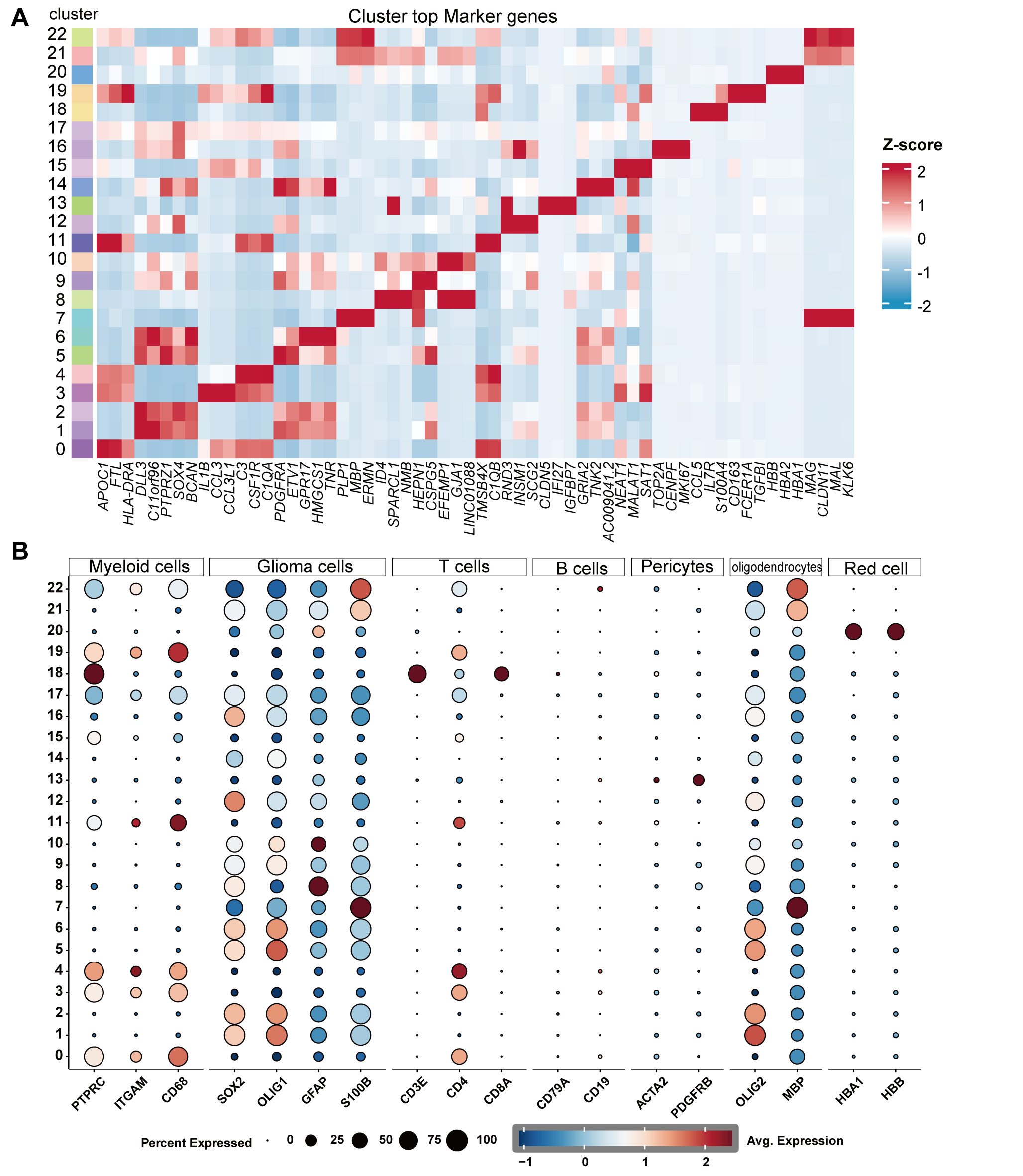

Supplement: Supplementary file 3 — Supplementary material 3. [file 12672_2024_1114_MOESM3_ESM.tif]
